# Left atrial reservoir strain combined with E/E' as a better single measure to predict elevated LV filling pressures in patients with coronary artery disease

**DOI:** 10.1186/s12947-020-00192-4

**Published:** 2020-04-25

**Authors:** Jingru Lin, Hong Ma, Lijian Gao, Yang Wang, Jiangtao Wang, Zhenhui Zhu, Kunjing Pang, Hao Wang, Weichun Wu

**Affiliations:** 1 Department of Echocardiography, State Key Laboratory of Cardiovascular Disease, Fuwai Hospital, National Center for Cardiovascular Diseases, Chinese Academy of Medical Sciences and Peking Union Medical College, 167 Beilishi Road, XiCheng District, Beijing, 100037 People’s Republic of China; 2 Department of Cardiology, Jiangsu Province Hospital, the First Affiliated Hospital of Nanjing Medical University, Nanjing, 210029 People’s Republic of China; 3 Department of Cardiology, State Key Laboratory of Cardiovascular Disease, Fuwai Hospital, National Center for Cardiovascular Diseases, Chinese Academy of Medical Sciences and Peking Union Medical College, Beijing, 100037 People’s Republic of China; 4 GE Healthcare, Beijing, 100176 People’s Republic of China; 5 Key Laboratory of Cardiovascular Imaging (Cultivation), Chinese Academy of Medical Sciences, Beijing 100037, People’s Republic of China

**Keywords:** Left atrial strain, Left ventricle, Diastolic function, Filling pressure, Coronary artery disease

## Abstract

**Background:**

The 2016 guidelines for left ventricular diastolic dysfunction diagnosis has been simplified from previous versions; however, multiparametric diagnosis approach still exists indeterminate left ventricular diastolic dysfunction category. Left atrial (LA) strain was recently found useful to predict elevated left ventricular (LV) filling pressures noninvasively. This study aimed to (1) analyze the diagnostic value of LA strain for noninvasive assessment of LV filling pressures in patients with stable coronary artery disease (CAD) with preserved LV ejection fraction (LVEF), using invasive hemodynamic assessment as the gold standard, and (2) explore whether LA strain combined with conventional diastolic parameters could detect elevated LV filling pressures alone.

**Methods:**

Sixty-four patients with stable CAD having LVEF > 50% and 30 healthy controls were enrolled. Two-dimensional speckle-tracking echocardiography was used to measure LA strain during the reservoir (LASr), conduit, and contraction phases. LV end-diastolic pressure (LVEDP), as a surrogate for LV filling pressures, was invasively obtained by left heart catheterization. Logistic regression was used to calculate the odds ratio to predict LV filling pressures. Pearson’s correlation was used to analyze associations between echocardiographic parameters and LVEDP. The area under the receiver-operating characteristic curve was calculated to determine the capability of the echocardiographic parameters to detect elevated LVEDP. Inter-technique agreement was analyzed by contingency tables and tested by kappa statistics.

**Results:**

LASr and the ratio of early-diastolic transmitral flow velocity (E) to tissue Doppler early-diastolic septal mitral annular velocity (E/E′septal) significantly predicted elevated LV filling pressures. LASr was combined with E/E′septal to generate a novel parameter (LASr/E/E′septal). LASr/E/E′septal had the best predictive ability of elevated LV filling pressures. LVEDP was negatively correlated with LASr and LASr/E/E′septal but positively correlated with E/E′septal. The area under the receiver-operating characteristic curve of LASr/E/E′septal was higher than that of LASr alone (0.83 vs. 0.75), better than all conventional LV diastolic parameters. Inter-technique agreement analysis showed that LASr/E/E′septal had good agreement with the invasive LVEDP measurement, better than the 2016 guideline (kappa = 0.63 vs. 0.25).

**Conclusions:**

LASr provided additive diagnostic value for the noninvasive assessment of LV filling pressures. LASr/E/E′septal had the potential to be a better single noninvasive index to predict elevated LV filling pressures in patients with stable CAD and preserved LVEF.

## Background

The American Society of Echocardiography (ASE) and European Association of Cardiovascular Imaging (EACVI) 2016 guideline algorithms for left ventricular diastolic dysfunction (LVDD) diagnosis are simpler than previous versions, making clinical use more convenient. The latest guideline, however, still cannot solve the diagnostic quandary of “indeterminate” status for patients whose data do not neatly fulfill the algorithms [1, 2]. Multiple studies have revealed that coronary artery disease (CAD) can cause remodeling of the left ventricular (LV) structure, leading to an adverse impact on LV relaxation and myocardial stiffness [3]. The resultant decreased LV relaxation and increased LV chamber stiffness then increase cardiac filling pressures, resulting in LVDD that often precedes systolic dysfunction [4, 5]. Abundant evidence demonstrates that patients with CAD have a high incidence of LVDD, leading to a lower long-term survival rate and worse prognosis [6, 7]. Thus, it is essential to estimate LV diastolic function earlier and more accurately in patients with CAD because it may substantially influence the cardiovascular outcome and choice of therapeutic strategy [8, 9]. However, in clinical practice, we found that a large population of patients with CAD fell into the indeterminate LVDD category, which was not conducive to early diagnosis and effective intervention. Therefore, an accurate parameter to detect LVDD earlier is in urgent need.

Recent studies have shown that abnormalities in the left atrium have an important effect on the pathophysiology and disease progression of LVDD and heart failure with preserved ejection fraction (HFpEF) [10–14]. Moreover, several studies have implied that left atrial (LA) strain, especially LA reservoir strain (LASr), is clinically useful for the noninvasive assessment of LV filling pressures [15–17].

However, in patients with CAD with preserved LV ejection fraction (LVEF), the role of LA strain when assessing LV filling pressures is unknown. Whether combining LA strain with conventional diastolic parameters could be a better single noninvasive method of predicting LVDD needs to be explored and compared with invasive hemodynamic data.

## Methods

### Study population

The study population included 64 patients treated at Fuwai Hospital from October 2013 to July 2014 for stable CAD. The patients were in sinus rhythm with normal resting wall motion and preserved LVEF (> 50%) having cardiac symptoms such as angina, ischemic-type chest pain, or other symptoms suggestive of myocardial ischemia. Thirty sex- and age-matched healthy controls with good acoustic windows were enrolled. All patients underwent left ventriculography and coronary angiography. Cardiac catheterization and echocardiography were performed on the same day (within 24 h). CAD was defined as > 50% luminal stenosis in one or more major epicardial vessels by visual assessment. The exclusion criteria were as follows: (1) patients with an LVEF ≤50%, including unstable conditions such as cardiac shock and ventricular aneurysm; (2) old myocardial infarction (within the past 3 months) or with distinct regional wall motion abnormalities at rest; (3) severe aortic or mitral disease; (4) atrial fibrillation, flutter, or ventricular-paced rhythm; and (5) hypermobile interatrial septum or interatrial septal aneurysm. The following data were gathered: clinical baseline characteristics, parameters of conventional echocardiography and two-dimensional speckle-tracking echocardiography (2D-STE), and hemodynamic data during left heart catheterization.

### Conventional transthoracic echocardiography

All conventional transthoracic echocardiographic measurements were performed at rest in the left lateral decubitus position using the Vivid E9 ultrasound system (GE Healthcare, Horten, Norway). Digital loops were stored and analyzed offline using EchoPAC clinical workstation software, version 202 (GE Healthcare). Measurements were performed on four consecutive heartbeats, and the average of three measurements was taken. Apical four- and two- chamber views were used to analyze the LV and LA volumes. LV end-systolic and -diastolic volume and ejection fraction (EF) were assessed by the biplane Simpson method. LA maximal volume, LA minimal volume, and LA volume at the onset time of the P wave were also measured by the biplane Simpson method. LA maximal volume was divided by the body surface area to obtain the LA maximal volume index (LAVI). The volumetric parameters of LA systolic function were calculated as follows [18]: LA emptying fraction (LAEF) = [(LA maximal volume − LA minimal volume)/LA maximal volume] × 100; LAEF-active = [(LA volume at the onset time of the P wave − LA minimal volume)/LA volume at the onset time of the P wave] × 100; LAEF-passive = [(LA maximal volume − LA volume at the onset time of the P wave)/LA maximal volume] × 100. LV mass was calculated as {0.8 × 1.04 ×[ (LVEDd + PWTd + SWTd)^3^ − (LVEDd)^3^] + 0.6}, where LVEDd is LV end-diastolic diameter, PWTd is end-diastolic posterior wall thickness, and SWTd is end-diastolic septal wall thickness. LV mass was then indexed for body surface area to generate the LV mass index (LVMI). Peak early-diastolic (E) and peak late-diastolic (A) transmitral velocities, the E/A ratio, and the deceleration time of the E wave were measured from the apical four-chamber view by pulsed Doppler echocardiography. Peak velocity of the tricuspid regurgitant jet was determined by continuous-wave Doppler. Peak early-diastolic myocardial velocity (E′) was measured from the apical four-chamber view by tissue Doppler echocardiography, at the levels of the basal portion of the septal and lateral mitral annuli, to generate E′septal and E′lateral, respectively, and the mean early-diastolic myocardial velocity (E′mean) was calculated. The ratio of early-diastolic transmitral flow velocity to tissue Doppler early-diastolic septal mitral annular velocity (E/E′septal), the ratio of early-diastolic transmitral flow velocity to tissue Doppler early-diastolic lateral mitral annular velocity (E/E′lateral), and the ratio of early-diastolic transmitral flow velocity to tissue Doppler mean early-diastolic myocardial velocity (E/E′mean) were also calculated. Mitral regurgitation severity was assessed using the Doppler quantitative technique, and according to the ASE guideline [19]. Conventional echocardiographic analysis including two-dimensional and Doppler imaging were performed by a single expert echocardiographer blinded to clinical information, invasive left ventricular pressure measurements, and 2D-STE results. Abnormal values for conventional LV diastolic parameters were determined according to the criteria of the latest (2016) guideline for LVDD [1]: (1) septal E' < 7 cm/s or lateral E' < 10 cm/s; (2) mitral average septal-lateral E/E' ratio (E/E' mean) > 14; (3) LAVI > 34 ml/m^2^ (using the biplane Simpson method); and (4) tricuspid regurgitation (TR) peak velocity >  2.8 m/s. Patients were diagnosed with LVDD when > 50% of the aforementioned criteria were positive and normal LV diastolic function when < 50% of these criteria were positive. In addition, when only 50% of the criteria were positive, patients were diagnosed as having indeterminate LV diastolic function. The normal reference values of other diastolic function parameters refer to the Normal Reference Ranges for Echocardiography (NORRE) Study [20].

### Two-dimensional speckle-tracking echocardiography

**Fig. 1:**
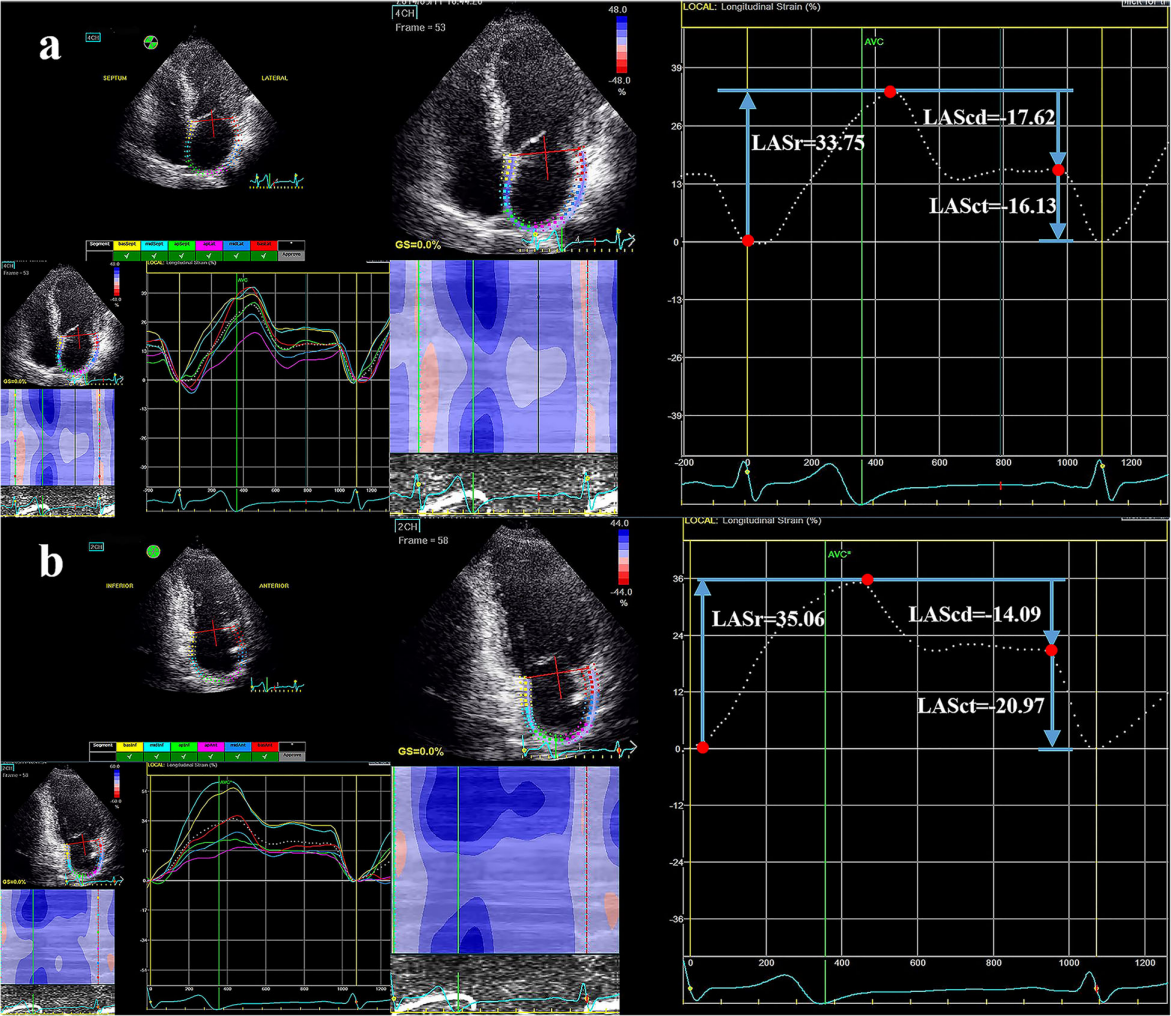
Representative example of two-dimensional STE–derived left atrial strain measurements during the reservoir, conduit, and contraction phases using an apical four-chamber view (**a**) and apical two-chamber view (**b**). Three measurement points (red dots) were used to calculate the values of deformation during the three phases. STE, speckle-tracking echocardiography

2D-STE image acquisition using the Vivid E9 ultrasound system (GE Healthcare) was performed with apical four-, three-, and two-chamber views to achieve optimal imaging quality for subsequent analyses. EchoPAC software was used to trace the LA endocardial border in both the four- and two- chamber views (LA strain values using the four-chamber view alone were analyzed and presented in the additional file 1), while taking care to exclude the appendage and pulmonary veins from the LA cavity [21]. LA longitudinal strain was calculated as the average LA strain in six segments. LASr, LA conduit strain (LAScd), and LA contraction strain (LASct) were used to represent the LA strain during the reservoir, conduit, and contraction phases, respectively. The reference point for zero strain was set at LV end-diastole. As the atrial wall lengthens during the reservoir phase, the strain in this phase is reported as a positive value, while the shortening of the LA wall during the other two phases suggests that they should be characterized by negative values (Fig. 1). LV global longitudinal strain (LVGLS) was measured from the apical four-, three-, and two-chamber views according to the EACVI/ASE recommendations [22]. The 2D-STE measurements and analyses were performed by a second experienced echocardiographer blinded to clinical information, invasive left ventricular pressure measurements, and conventional transthoracic echocardiographic findings. All echocardiographic measurements and analyses were the average of three consecutive cycles.

### Invasive LV pressure measurements

Left heart catheterization was performed through the radial artery by an expert interventional cardiologist who was blinded to the echocardiographic data. Before coronary angiography, transducers were balanced prior to the acquisition of hemodynamic data with zero level at the midaxillary line [23]. A 6 F pigtail catheter was placed at the mid-LV cavity for LV end-diastolic pressure (LVEDP) measurements. LVEDP was measured at the R wave on the electrocardiogram, and finally calculated as the mean value of four consecutive heart cycles during quiet respiration [24]; LVEDP was determined at end-expiration and was considered elevated if greater than 15 mmHg [25].

### Intra- and inter-observer variabilities

The reproducibility of LA strain measurements was assessed in 15 randomly selected patients. Intra-observer agreement was assessed using measurements made by the same observer on the same echocardiographic images in random order at an average interval of 2 weeks. Inter-observer agreement was evaluated by comparing the measurements of the first observer with those of a second independent observer; both observers were blinded to previous measurements and were unaware of clinical data.

### Statistical analysis

Continuous variables were presented as the mean ± standard deviation and were analyzed with an independent *t*-test. Categorical data were presented as absolute numbers or percentages and were analyzed with the chi-squared test or Fisher’s exact test. Logistic regression was used to calculate the odds ratio to predict LV filling pressures. Pearson’s correlation was used to analyze associations between echocardiographic parameters and LVEDP. The area under the receiver-operating characteristic (ROC) curve (AUC) was calculated to determine the capability of the echocardiographic parameters to detect elevated LVEDP [26]. Contingency tables of normal and elevated pressure values assessed by echocardiographic techniques and the invasive reference technique were created to evaluate inter-technique agreement, which was tested using kappa statistics.

Reproducibility was assessed by Bland-Altman plots, intraclass correlation coefficients, and the calculation of the absolute differences between two measurements divided by the mean, which were expressed as percentages.

Analyses were performed using SPSS, version 25.0 (IBM Corp., Armonk, NJ, USA), MedCalc, version 19.0.5 (MedCalc Software, Ltd., Ostend, Belgium), and GraphPad Prism 8 (GraphPad Software, San Diego, CA, USA). Two-tailed *P*-values of < 0.05 were considered to indicate statistical significance.

## Results

### Baseline characteristics

Sixty patients with stable CAD and preserved LVEF were included in this study. Four patients with poor image quality in more than one LA segment were excluded (feasibility 93.8%). The control group comprised 30 age- and sex-matched healthy participants. Demographic and clinical characteristics are presented in Table 1.

**Table 1:** Baseline Characteristics, Catheterization Data, and Echocardiographic Variables

Characteristic/ Variable	Control (*n* = 30)	All Patients With CAD (*n* = 60)	Patients With CAD		*P* Value, Control vs. CAD	*P* Value, group I vs. group II
			LVEDP≤15 mmHg (group I, *n* = 27)	LVEDP>15 mmHg (group II, *n* = 33)		
Baseline Characteristics						
Age, y	53 ± 7	56 ± 9	58 ± 8	55 ± 10	0.19	0.19
Male, n (%)	20(66.7)	48(80.0)	21(77.8)	27(81.8)	0.72	0.70
HR, beats/min	64 ± 9	66 ± 9	67 ± 10	66 ± 8	0.25	0.60
Systolic BP, mmHg	119 ± 10	128 ± 20	127 ± 24	128 ± 16	0.02	0.79
Diastolic BP, mmHg	74 ± 5	79 ± 11	77 ± 10	80 ± 12	0.00	0.30
BMI, kg/m^2^	25.0 ± 1.5	25.8 ± 2.8	25.7 ± 2.9	25.8 ± 2.8	0.14	0.87
Medical history, n (%)						
Diabetes	–	30(50.0)	13(48.1)	17(51.5)	–	0.41
Hypertension	–	39(65.0)	18(66.7)	21(63.6)	–	0.81
Dyslipidemia	–	53(88.3)	24(88.9)	29(87.9)	–	0.74
Prior MI	–	26(43.3)	12(44.4)	14(42.4)	–	0.66
Mild MR	–	10(16.7)	4(14.8)	6(18.2)	–	0.96
≥ Moderate MR	–	1(1.7)	–	1(3.0)	–	–
CAD: vessel involved, n (%)						
Single vessel	–	16(26.7)	7(25.9)	9(27.3)	–	0.91
Multiple vessels	–	44(73.3)	20(74.1)	24(72.7)	–	0.91
Culprit vessel, n (%)						
LMCA	–	8(13.3)	3(11.1)	5(15.2)	–	0.94
LAD	–	54(90.0)	23(85.2)	31(93.9)	–	0.49
RCA	–	38(63.3)	17(63.0)	21(63.6)	–	0.96
LCx	–	40(66.7)	19(70.4)	21(63.6)	–	0.58
Coronary dominance, n (%)						
Right	–	51(85.0)	23(85.2)	28(84.8)	–	1.00
Left	–	5(8.3)	2(7.4)	3(9.1)	–	1.00
Balanced	–	4(6.7)	2(7.4)	2(6.1)	–	1.00
Medication, n (%)						
β-Blockers	–	52(86.7)	24(88.9)	28(84.8)	–	0.94
Antiplatelet drugs	–	60(100.0)	27(100.0)	33(100.0)	–	–
ACEI/ARBs	–	55(91.7)	25(92.6)	30(90.9)	–	1.00
CCBs	–	32(53.3)	15(55.6)	17(51.5)	–	0.76
Loop diuretics	–	16(26.7)	6(22.2)	10(30.3)	–	0.48
Statins	–	56(93.3)	25(92.6)	31(93.9)	–	1.00
PCI, n (%)	–	38(63.3)	17(63.0)	21(63.6)	–	0.96
CABG, n (%)	–	10(16.7)	5(18.5)	5(15.2)	–	1.00
Catheterization Data						
LVEDP, mmHg	–	18.9 ± 7.8	11.9 ± 2.4	24.6 ± 5.8	–	0.00
Conventional Echocardiographic Variables						
TR peak velocity > 2.8 m/s, n (%)	–	5(8.3)	1(3.7)	4(12.1)	–	0.48
Mitral E, cm/s	75.3 ± 12.1	79.4 ± 15.9	79.6 ± 20.2	79.3 ± 11.7	0.29	0.95
Mitral A, cm/s	65.3 ± 14.9	71.7 ± 16.8	69.9 ± 15.5	73.2 ± 17.9	0.13	0.46
Mitral E/A ratio	1.21 ± 0.34	1.16 ± 0.33	1.17 ± 0.33	1.15 ± 0.34	0.57	0.78
DT, ms	166 ± 20	178 ± 21	178 ± 21	177 ± 21	0.03	0.87
E′septal, cm/s	9.2 ± 1.8	6.7 ± 1.5	7.2 ± 1.6	6.2 ± 1.3	0.00	0.01
E′lateral, cm/s	12.3 ± 2.2	9.4 ± 2.7	9.9 ± 2.8	9.0 ± 2.5	0.00	0.18
E′mean, cm/s	10.8 ± 2.0	8.1 ± 1.9	8.6 ± 2.0	7.6 ± 1.7	0.00	0.052
E/E′septal	8.4 ± 1.6	12.3 ± 3.0	11.3 ± 2.9	13.1 ± 2.9	0.00	0.02
E/E′lateral	6.3 ± 1.3	9.0 ± 2.7	8.4 ± 2.6	9.4 ± 2.8	0.00	0.18
E/E′mean	7.1 ± 1.4	10.3 ± 2.6	9.6 ± 2.4	10.8 ± 2.6	0.00	0.04
LAEF (%)	71.2 ± 8.7	67.3 ± 6.5	69.1 ± 5.7	65.8 ± 6.8	0.04	0.05
LAEF-active (%)	50.8 ± 9.5	51.4 ± 8.0	53.6 ± 6.7	49.7 ± 8.6	0.78	0.06
LAEF-passive (%)	40.7 ± 15.7	32.4 ± 9.0	33.1 ± 9.7	31.8 ± 8.5	0.03	0.56
LAVI, ml/m^2^	22.1 ± 8.3	31.7 ± 5.7	30.1 ± 4.7	33.0 ± 6.2	0.00	0.04
LVMI,g/m^2^	75.4 ± 22.0	103.5 ± 24.9	102.9 ± 27.4	104.0 ± 23.2	0.00	0.88
LVEF, %	62.8 ± 3.1	61.4 ± 3.1	61.6 ± 3.2	61.2 ± 2.9	0.09	0.66
2D-STE Variables						
LVGLS (%)	−20.2 ± 1.6	−19.0 ± 1.8	−19.5 ± 1.8	−18.6 ± 1.7	0.01	0.07
LASr (%)	28.2 ± 4.0	23.0 ± 5.1	25.6 ± 5.4	20.9 ± 3.7	0.00	0.00
LAScd (%)	−15.2 ± 4.0	−10.5 ± 3.7	−11.5 ± 4.1	−9.6 ± 3.2	0.00	0.06
LASct (%)	−13.0 ± 2.2	−12.4 ± 3.5	−13.9 ± 3.9	−11.2 ± 2.6	0.45	0.00
LASr/E/E′septal(%)	3.6 ± 1.3	2.0 ± 0.6	2.4 ± 0.6	1.7 ± 0.5	0.00	0.00

*ACEI* Angiotensin-converting enzyme inhibitors; *ARBs* Angiotensin II receptor blockers;

*BMI* Body mass index; *BP* Blood pressure; *CAD* Coronary artery disease; *CCBs* Calcium channel blockers; *CABG* Coronary artery bypass graft; *DT* Deceleration time; *E/E′septal* Ratio of early-diastolic transmitral flow velocity to tissue Doppler early-diastolic septal mitral annular velocity *HR* Heart rate; *LAD* Left anterior descending artery; *LAEF* Left atrial total emptying fraction; *LAEF-active* Left atrial active emptying fraction; *LAEF-passive* Left atrial passive emptying fraction; *LAScd* Left atrial conduit strain; *LASct* Left atrial contraction strain; *LASr* LA reservoir strain; *LAVI* LA maximal volume index; *LCx* Left circumflex artery; *LMCA* Left main coronary artery; *LVEDP* Left ventricular end-diastolic pressure; *LVEF* Left ventricular ejection fraction; *LVGLS* Left ventricular global longitudinal strain; *LVMI* Left ventricular mass index; *MI* Myocardial infarct; *MR* Mitral regurgitation; *PCI* percutaneous coronary intervention; *RCA* Right coronary artery; *TR* Tricuspid regurgitation; *2D-STE* two-dimensional speckle-tracking echocardiography

The mean age of the 60 patients with CAD was 56 ± 9 years, and most (48 patients) were men. Overall, 30 patients had diabetes mellitus, 39 had hypertension, 53 had dyslipidemia, and 26 had a prior myocardial infarct. On coronary angiography, coronary artery diameter stenosis > 50% was present in a single vessel in 16 (26.7%) patients, and two or three (multiple) vessels in 44 (73.3%) patients. The location of the culprit lesion was the left main coronary artery in eight (13.3%) patients, the left anterior descending coronary artery in 54 (90.0%) patients, the left circumflex coronary artery in 40 (66.7%) patients, and the right coronary artery in 38 (63.3%) patients. The types of coronary dominance were right, left, and balanced in 51 (85.0%), five (8.3%), and four (6.7%) patients, respectively. Compared with the control group, the CAD group had higher systolic and diastolic blood pressures. Other baseline characteristics did not significantly differ between these two groups (Table 1).

The CAD group was further divided into group I (LVEDP ≤15 mmHg, *n* = 27) and group II (LVEDP > 15 mmHg, *n* = 33). No significant differences were found between group I and group II in terms of age, gender, blood pressures, medical history, medication, or coronary angiography findings (Table 1).

### Conventional echocardiographic parameters

Compared with the CAD group, the control group had significantly higher peak early- diastolic myocardial velocities, LAEF and LAEF-passive, and significantly lower deceleration time, LAVI, LVMI, E/E′septal, E/E′lateral, and E/E′mean. Compared with group I, group II had a significantly lower E′septal, E′mean, and LAEF, and a higher LAVI, E/E′septal, and E/E′mean. However, there was no significant difference in the LVMI between these two subgroups (Table 1).

### 2D-STE parameters

Compared with the control group, the CAD group had significantly lower LVGLS, LASr, and LAScd. Compared with group I, group II had significantly lower LASr and LASct (Table 1).

### Logistic regression analysis and Pearson’s correlation analysis

In univariate logistic regression analysis, the variables that significantly predicted elevated LV filling pressures included LASr, LASct, LAVI, E′septal, E/E′septal, and E/E′mean. LAScd, LVGLS, LAEF, and LAEF-active did not show significant predictive value in detecting LVDD (Table 2). In multivariate logistic regression analysis, the variables that significantly predicted elevated LV filling pressures were LASr (OR = 0.75; *P* = 0.003), and E/E′septal (OR = 1.27; *P* = 0.043) (Table 2). Then LASr was combined with E/E′septal to generate a novel parameter (LASr/E/E′septal). LASr/E/E′septal was significantly lower in the CAD group, and it was lower in group II than in group I (Table 1). When including LASr/E/E′septal in the multivariate logistic regression analysis, LASr/E/E′septal (OR = 0.08; *P* = 0.000) became the only parameter to significantly predict elevated LV filling pressures.

**Table 2:** Logistic Regression Analysis of Variables Indicating Left Ventricular Diastolic Dysfunction

Variable	Univariate analysis			Multivariate analysis		
	OR	95% CI	P	OR	95% CI	P
LASr(%)	0.76	0.64–0.91	0.00	0.75	0.62–0.91	0.00
LAScd (%)	1.15	0.99–1.35	0.07			
LASct(%)	1.32	1.09–1.60	0.01			
LVGLS (%)	1.33	0.97–1.83	0.08			
LAEF (%)	0.912	0.83–1.00	0.06			
LAEF-active (%)	0.935	0.87–1.00	0.07			
LAVI, ml/m^2^	1.12	0.99–1.26	0.02			
E′septal, cm/s	0.63	0.43–0.92	0.02			
E′lateral, cm/s	0.87	0.71–1.07	0.19			
E′mean, cm/s	0.75	0.56–1.01	0.06			
E/E′septal	1.30	1.03–1.63	0.03	1.27	1.01–1.61	0.04
E/E′mean	1.24	0..99–1.56	0.06			
LASr/E/E′septal(%)	0.08	0.02–0.31	0.00			

CI Confidence interval; *OR* Odds ratio; for other abbreviations, see Table 1

**Fig. 2:**
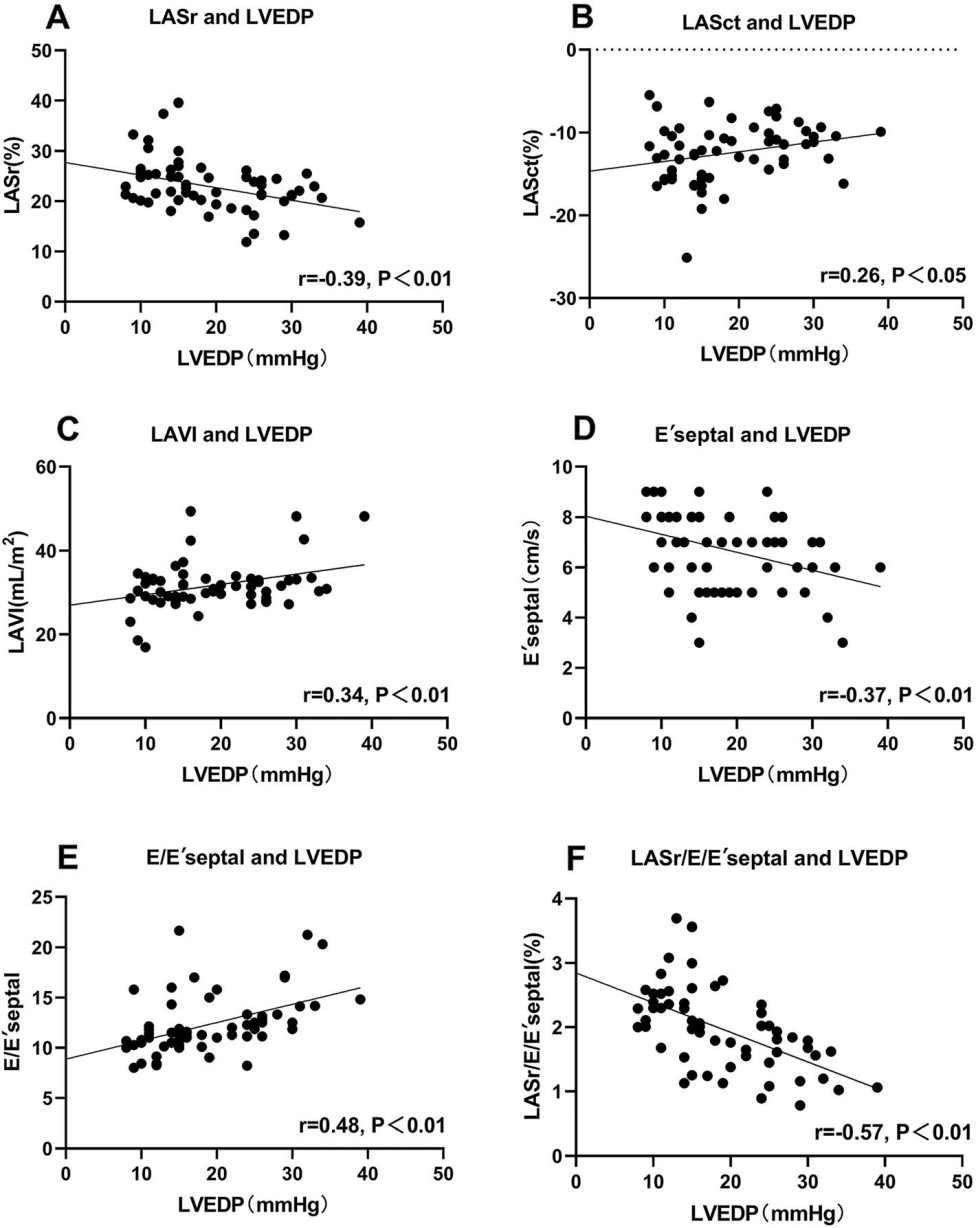
Correlation analyses of echocardiographic parameters and LVEDP. LASct = left atrial contraction strain; LASr = left atrial reservoir strain; LVEDP = left ventricular end-diastolic pressure

Pearson’s correlation analysis revealed that LVEDP was positively correlated with E/E′septal (*r* = 0.48, *P* < 0.01) and negatively correlated with LASr (*r* = − 0.39, *P* < 0.01) and LASr/E/E′septal (*r* = − 0.57, *P* < 0.01) (Fig. 2).

### ROC curve analysis

**Fig. 3:**
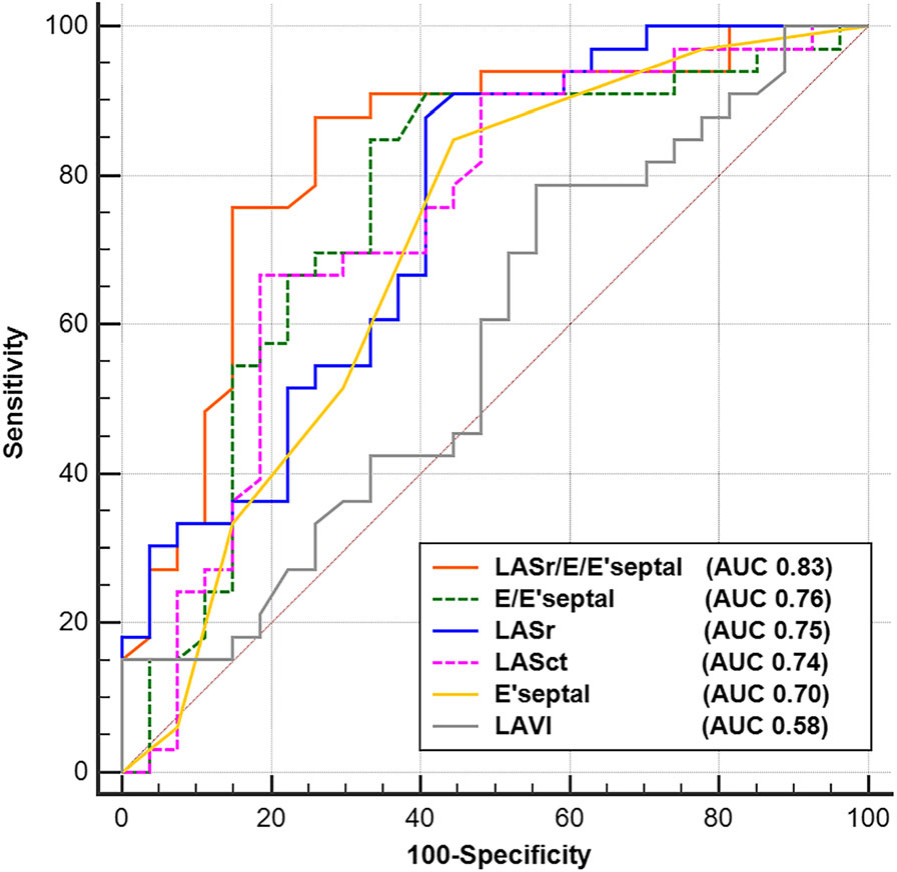
Receiver operating characteristic curve analysis. LASr = left atrial reservoir strain

ROC curve analysis showed that LASr could predict elevated LVEDP in patients with CAD and preserved LVEF (AUC = 0.75; 95% CI, 0.62–0.85), and a cut-off value of LASr < 24.7% was able to most accurately identify patients with LVEDP > 15 mmHg. However, LASr/E/E′septal was superior to LASr alone, with an AUC of 0.83 (Table 3 and Fig. 3).

**Table 3:** Receiver Operating Characteristic Curve Analysis

Variable	AUC [95% CI]	*P*-value	Cutoff	Sensitivity (%)	Specificity (%)
LASr(%)	0.75[0.62–0.85]	0.00	24.7	87.9	59.3
LASct(%)	0.74[0.61–0.84]	0.00	−11.6	66.7	81.5
LAVI, ml/m^2^	0.58[0.44–0.70]	0.31	29.2	78.8	44.4
E′septal, cm/s	0.70[0.57–0.81]	0.00	7	84.9	55.6
E/E′septal	0.76[0.63–0.86]	0.00	11.1	84.9	66.7
LASr/E/E′septal(%)	0.83[0.71–0.92]	0.00	2.1	87.9	74.1

*AUC* Area under the curve; CI Confidence interval; for other abbreviations, see Table 1

### Consistency of echocardiographic and invasive techniques

**Fig. 4:**
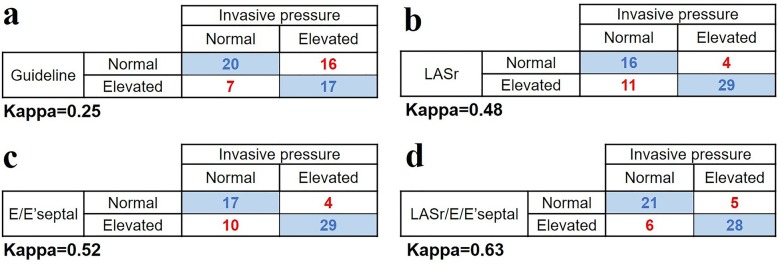
Contingency tables of agreement among the 2016 ASE Diastolic Guidelines **a**. Left atrial strain parameter **b**. Conventional echocardiographic parameter **c** and new combinational echocardiographic parameter **d**. The kappa coefficient for each parameter is listed below each contingency table. ASE = American Society of Echocardiography; LASr = left atrial reservoir strain

The agreement between the 2016 diastolic function guideline algorithms and the reference technique in our study population was slightly low. After we classified the patients with indeterminate condition as LVDD, the kappa coefficient was only 0.25. The kappa coefficient for LASr was 0.48, showing substantial agreement with the reference technique. LASr/E/E′septal had a higher kappa coefficient (kappa = 0.63) than LASr, showing good agreement with the invasive technique (Fig. 4).

### Reproducibility of LA strain parameters

**Fig. 5:**
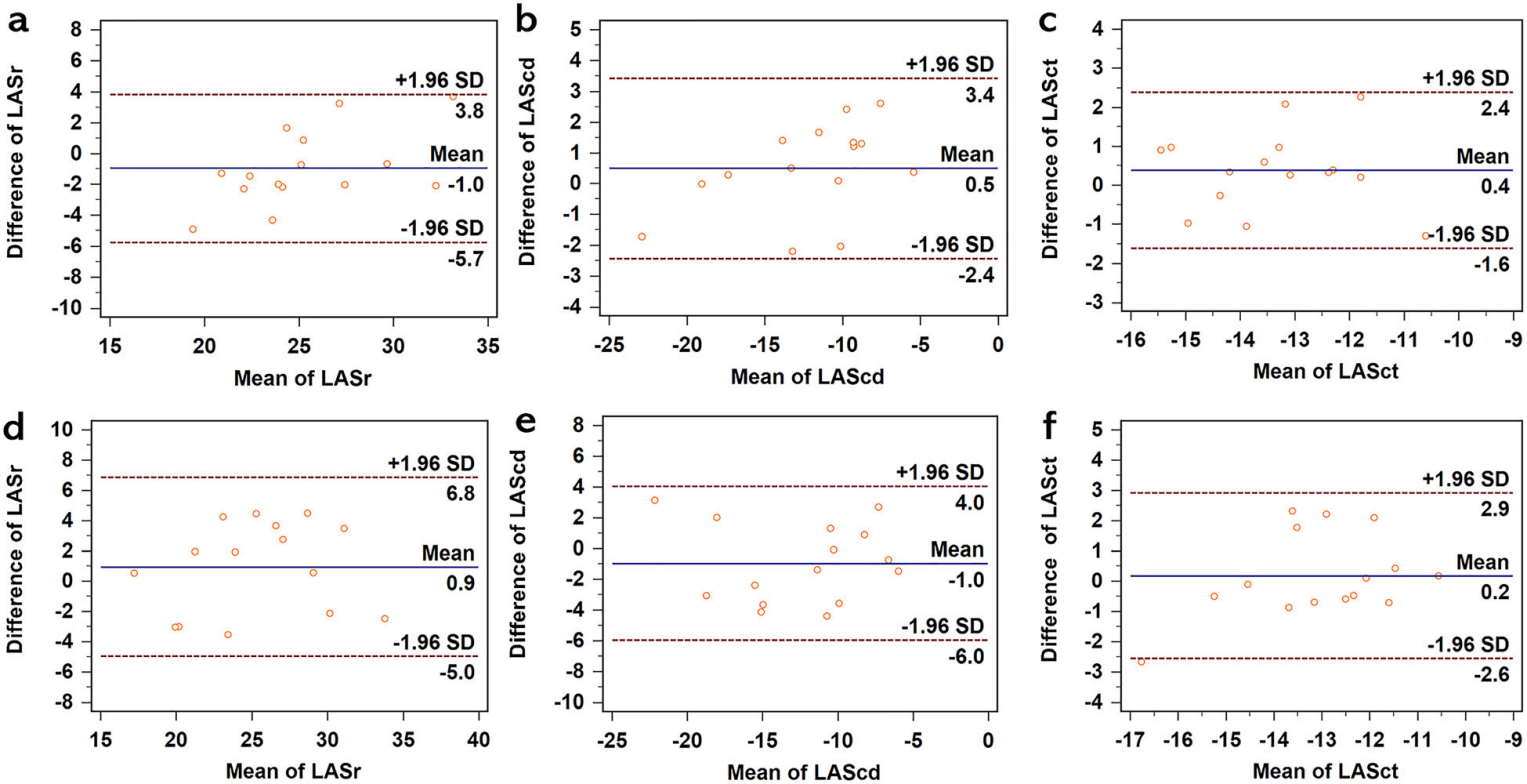
Bland-Altman analysis for intra- and inter-observer variabilities for LA strain measurements. **a**, **b**, **c** Intra-variabilities of LASr, LAScd, and LASct, respectively. **d**, **e**, **f** Inter-variabilities of LASr, LAScd, and LASct, respectively. LAScd = left atrial conduit strain; LASct = left atrial contraction strain; LASr = left atrial reservoir strain

The intra- and inter-observer agreements of LASr, LAScd, and LASct are summarized in Table 4 and Fig. 5.

**Table 4:** Intra- and Inter-observer Variabilities for LA Strain Parameters

	Intra-observer			Inter-observer		
LA strain parameters	Variability (%)	Intra-class correlation coefficient	95% CI	Variability (%)	Intra -class correlation coefficient	95% CI
LASr(%)	7.87 ± 6.77	0.90	0.69–0.97	11.20 ± 5.02	0.90	0.69–0.97
LAScd(%)	10.53 ± 10.08	0.97	0.90–0.99	17.60 ± 12.63	0.93	0.79–0.98
LASct(%)	6.93 ± 3.67	0.87	0.61–0.96	7.53 ± 8.00	0.81	0.43–0.94

For abbreviations see Tables 1 and 2

## Discussion

LA strain assessed by 2D-STE has now been evaluated in multiple conditions, such as heart failure, atrial fibrillation, and valvular diseases [16]. LA reservoir strain is considered to be an indicator of LA dysfunction and is reduced in the setting of LV diastolic dysfunction [17]. However, there are few research reports on the role of LA strain when predicting elevated LV filling pressures in patients with CAD. Until recently, an investigation has suggested that LA peak systolic strain may be a helpful and complementary method to evaluate diastolic function in patients with ST-elevation myocardial infarction [27].

In the present study, we estimated the diagnostic accuracy of LA strain in patients with stable CAD having preserved EF against invasive hemodynamic reference data. We demonstrated that LASr was available in the most patients and outperformed other commonly used echocardiographic parameters that have often been used in the evaluation of LVDD according to the latest guideline. More importantly, we found that the ratio of LASr to estimated LV filling pressures E/E'septal (LASr/E/E′septal) was a better stand-alone parameter to predict elevated LV filling pressures in patients with stable CAD who have preserved LVEF.

Our findings demonstrated that LASr (AUC = 0.75) was more beneficial in predicting elevated LVEDP and detecting LVDD than all the above mentioned conventional parameters except E/E′septal (AUC = 0.76). However, when countered with clinical situations in which the acquisition of Doppler parameters is complicated or the results are ambiguous, LASr has advantages over E/E′septal in the evaluation of LVDD. The results of our research were consistent with several previous studies, which indicated that LA reservoir strain decreased as LV diastolic dysfunction worsened [17] and provided incremental diagnostic information beyond LAVI alone [28].

LA strain reflects the LV diastolic function for the following reasons. In the early stages of LVDD, as LV pressures and/or stiffness increase, the left atrium may compensate for higher LV pressures by contracting, but prolonged higher LV pressures and dysfunction may cause LA dilatation [29, 30]. Several recent studies suggested that, in the setting of increased LV pressures, LA function is already compromised before the LA starts to dilate [31, 32]. In patients with a normally sized left atrium, these transmitted pressures gradually blunt the compliance of the atrium, impairing atrial relaxation. In these patients, the result is a reduction in the ability of the atrium to act as a reservoir in ventricular systole, eventually leading to LA dilatation and mechanical failure [33, 34]. LA strain globally reflects atrial function, remodeling, and distensibility that become progressively impaired in chronic LVDD, such as in patients with HFpEF [11, 14, 35]. However, the role of LA strain when assessing filling pressures in patients with reduced LVEF is still controversial. Melenovsky et al. and Singh et al. believed that LASr was less accurate in characterizing LV filling pressures in patients with LV systolic dysfunction than in patients with normal systolic function [12, 15]. In contrast, Cameli et al. demonstrated that in patients with advanced systolic heart failure, peak atrial longitudinal strain provided a better estimation of LV filling pressures [36].

Normalizing LA reservoir strain to the estimated filling pressure index (E/E′septal) further improved the discrimination ability (AUC = 0.83) of elevated LV filling pressures with relatively high sensitivity (87.9%) and specificity (74.1%) in patients with stable CAD who had preserved LVEF. LASr/E/E′septal was comprised of the best LA strain parameter and a preferable estimated LV filling pressures parameter(E/E′septal), revealing not only the LV function affected by LV filling pressures but also the LA compliance influenced by LV diastolic function, which was a more comprehensive index in detecting elevated LV filling pressures. In a recent study, Reddy et al. found that LA compliance (as estimated by LASr/E/E′septal) may be the single best echocardiographic correlate of elevated filling pressures either at rest or during exertion in patients with preserved LVEF [16], and our findings were partly consistent with this conclusion, while Reddy et al. studied a population of patients with HFpEF, a more serious condition than LVDD. Previous studies demonstrated that once heart failure with preserved EF has developed, no therapeutic intervention that has provided a prognostic impact has been identified to date [37]. However, preventive treatment strategies are effective prior to diagnosis of HFpEF [38]. Thus, the novel parameter (LASr/E/E′septal) found in our research fills an urgent need and has great clinical significance in distinguishing LVDD from the normal in case of developing into heart failure.

When the 2016 guideline algorithms were used to diagnose LVDD, 20 of 60 patients (33.3%) in our study were categorized as “indeterminate” status. Among the 20 patients with indeterminate results, LASr discriminated the filling pressures status in agreement with invasive measurements in 15 patients (75.0%), while LASr/E/E′septal correctly determined the status in 17 patients (85.0%). The findings suggested that LASr and LASr/E/E′septal added great complementary diagnostic value to the current guideline, particularly in patients diagnosed with “indeterminate diastolic function”. Meanwhile, LASr and LASr/E/E′septal showed substantial or good agreement with the invasive technique (kappa = 0.48 and kappa = 0.63, respectively), significantly better than that of the 2016 guideline (kappa = 0.25). Prior studies demonstrated that in the early stages of diastolic dysfunction, LVEDP is the only abnormally elevated pressure (because of a large atrial pressure wave), while mean pulmonary capillary wedge pressure and LA pressure remain normal at this time [1, 39]. However, the 2016 guideline algorithms are based on the prediction of mean pulmonary capillary wedge pressure but not LVEDP [1, 39]. To a certain extent, these findings may explain why 2016 guideline algorithms showed poor agreement with the invasive technique (LVEDP) in our study and why a great number of patients with CAD fell into the indeterminate LVDD category in clinical practice.

LASr provided additional value in predicting elevated LV filling pressures in patients with stable CAD and preserved EF in comparison with conventional echocardiographic parameters in the 2016 guideline, especially when encountered with “indeterminate diastolic dysfunction” or clinical situations in which the acquisition of Doppler parameters is difficult, such as lack of or incomplete tricuspid regurgitation jet, tachycardia obscuring mitral annular tissue Doppler tracing and so on. Furthermore, LA reservoir strain combined with E/E'septal (LASr/ E/E'septal) proved to be a better noninvasive parameter to predict elevated LV filling pressures and identify LVDD earlier and more accurately in patients with stable CAD and preserved EF. However, the routine clinical use of LV filling pressure assessment by LASr/ E/E’septal alone needs further validation in larger samples. In addition, further research is required to explore how best to incorporate LASr and LASr/E/E′septal into multiparametric diagnostic models for CAD patients with preserved LVEF, and to validate the optimal cutoff value for these parameters to differentiate LVDD from the normal.

Several limitations of our research should be noted. First, the sample size in our study was relatively small. Thus, further multicenter studies with large samples are necessary to confirm our preliminary findings. Second, patients with atrial fibrillation or other severe arrhythmia were excluded from this study; the populations comprised patients with CAD with preserved LVEF. Thus, the present findings may be only generalizable to patients with sinus rhythm with intermediate to high probability of LVDD and preserved LVEF. Third, we used the average of the four- and two-chamber views to analyze LA strain, rather than the four-chamber view alone as recommended [21]. However, both methods appeared to perform similarly, which was proved by a sub-analysis (see Additional files 1 and 2). Further validation of the utility of using only the four-chamber view for LA strain is needed in larger samples of patients with various diseases. Fourth, the results of coronary angiography were interpreted by an expert interventional cardiologist based on visual assessment, without performing fractional flow reserve or instant wave-frame ratio to confirm the hemodynamic relevance, which should be improved in our subsequent research. Fifth, EchoPAC is not dedicated software for the analysis of LA strain; currently, a dedicated software product recognized for the assessment of LA strain is not available, and thus we applied the commonly used software for the left ventricle in our study. Furthermore, the values of LA strain are vendor-dependent; therefore, the same 2D-STE software should be used to analyze LA strain for patient diagnosis and follow-up [21, 40]. Finally, the reliability of LA strain is influenced by image quality, and LA strain needs to be analyzed by ultrasound software offline. Therefore, the acquisition of LA strain may require skillful operators and consume more time than traditional echocardiographic parameters. But more and more studies have confirmed the feasibility of LA strain and provided normal values, which enable LA strain to be a useful tool for diastolic assessment in future clinical practice [41, 42].

## Conclusions

LASr could provide additional value in predicting elevated LV filling pressures in patients with stable CAD who have preserved EF in comparison with the conventional echocardiographic parameters of the 2016 guideline. LASr/E/E′septal, superior to LASr, had the potential to be a better stand-alone echocardiographic parameter to identify elevated LV filling pressures and discriminate LVDD earlier and more accurately in patients with stable CAD and preserved EF.

## Supplementary information

**Additional file 1: Table S1.** LA Strain Variables of four- and two-chamber views vs. four-chamber view alone. **Table S2.** Logistic Regression Analysis of Variables Indicating Left Ventricular Diastolic Dysfunction (including LA strain variables of four-chamber view alone). **Table S3.** Receiver Operating Characteristic Curve Analysis (including LA strain variables of four-chamber view alone).

**Additional file 2: Figure S1.** Bland-Altman analysis for intra-observer and inter-observer variabilities for LA strain measurements of four-chamber view alone.

## Data Availability

The datasets used and analyzed during the current study are not publicly available because they contain information that could compromise the privacy of research participants; however, the datasets are available from the corresponding author on reasonable request.

## Abbreviations

2D-STE: Two-dimensional speckle-tracking echocardiography
ASE: American society of echocardiography
AUC: Area under the curve
CAD: Coronary artery disease
DD: Diastolic dysfunction
E/E′septal: Ratio of early-diastolic transmitral flow velocity to tissue Doppler early-diastolic septal mitral annular velocity
EACVI: European association of cardiovascular imaging
EF: Ejection fraction
HFpEF: Heart failure with preserved ejection fraction
LA: Left atrial
LAEF: LA emptying fraction
LAScd: LA conduit strain
LASct: LA contraction strain
LASr: LA reservoir strain
LAVI: LA maximal volume index
LV: Left ventricular
LVEDP: LV end-diastolic pressure
LVGLS: LV global longitudinal strain
LVMI: LV mass index
NORRE: Normal reference ranges for echocardiography
OR: Odds ratio
ROC: Receiver-operating characteristic
