# Supplementary material for: Left atrial reservoir strain combined with E/E' as a better single measure to predict elevated LV filling pressures in patients with coronary artery disease
Source: Cardiovasc Ultrasound. 2020 Apr 25;18:11. doi: 10.1186/s12947-020-00192-4 (PMC7183713; doi:10.1186/s12947-020-00192-4)
Supplement: Supplementary file 1 — Additional file 1: Table S1. LA Strain Variables of four- and two-chamber views vs. four-chamber view alone. Table S2. Logistic Regression Analysis of Variables Indicating Left Ventricular Diastolic Dysfunction (including LA strain variables of four-chamber view alone). Table S3. Receiver Operating Characteristic Curve Analysis (including LA strain variables of four-chamber view alone). [file 12947_2020_192_MOESM1_ESM.docx]

**Supplementary Table 1** LA Strain Variables of four- and two-chamber views vs. four-chamber view alone

| Characteristic/ Variable | Control  (n=30) | All Patients With CAD  (n=60) | Patients With CAD | | P Value，  Control vs. CAD | P Value，  group I vs. group II |
| --- | --- | --- | --- | --- | --- | --- |
|  |  |  | LVEDP≤15mmHg  （group I，n=27） | LVEDP＞15mmHg  (group II，n=33) |  |  |
| LASr-4C (%) | 27.0±6.1 | 21.7±6.2 | 24.7±7.1 | 19.3±4.1 | 0.00 | 0.00 |
| LAScd-4C (%) | -14.0±5.3 | -9.6±4.1 | -10.5±4.5 | -8.9±3.6 | 0.00 | 0.13 |
| LASct-4C (%) | -12.7±3.3 | -12.2±4.9 | -14.1±5.5 | -10.6±3.7 | 0.63 | 0.01 |
| LASr (%) | 28.2±4.0 | 23.0±5.1 | 25.6±5.4 | 20.9±3.7 | 0.00 | 0.00 |
| LAScd (%) | -15.2±4.0 | -10.5±3.7 | -11.5±4.1 | -9.6±3.2 | 0.00 | 0.06 |
| LASct (%) | -13.0±2.2 | -12.4±3.5 | -13.9±3.9 | -11.2±2.6 | 0.45 | 0.00 |
| LASr/E/E′septal（%） | 3.6±1.3 | 2.0±0.6 | 2.4±0.6 | 1.7±0.5 | 0.00 | 0.00 |
| LASr/E/E′septal-4C（%） | 3.4±1.6 | 1.9±0.7 | 2.3±0.7 | 1.5±0.4 | 0.00 | 0.00 |

LASr-4C = left atrial reservoir strain of four-chamber view; LAScd-4C = left atrial conduit strain of four-chamber view; LASct-4C = left atrial contraction strain of four-chamber view; LASr = left atrial reservoir strain; LAScd = left atrial conduit strain; LASct = left atrial contraction strain.

**Supplementary Table 2** Logistic Regression Analysis of Variables Indicating Left Ventricular Diastolic Dysfunction

| Variable | Univariate analysis | | |  | Multivariate analysis | | |  |
| --- | --- | --- | --- | --- | --- | --- | --- | --- |
|  | OR | 95% CI | P |  | OR | 95% CI | P |  |
| LASr（%） | 0.76 | 0.64-0.91 | 0.00 |  | 0.75 | 0.62-0.91 | 0.00 |  |
| LAScd (%) | 1.15 | 0.99-1.35 | 0.07 |  |  |  |  |  |
| LASct（%） | 1.32 | 1.09-1.60 | 0.01 |  |  |  |  |  |
| LASr-4C (%) | 0.83 | 0.73-0.94 | 0.00 |  |  |  |  |  |
| LASct-4C (%) | 1.19 | 1.05-1.36 | 0.01 |  |  |  |  |  |
| E/E′septal | 1.30 | 1.03-1.63 | 0.03 |  | 1.27 | 1.01-1.61 | 0.04 |  |
| LASr/E/E′septal（%） | 0.08 | 0.02-0.31 | 0.00 |  |  |  |  |  |

CI = confidence interval; OR = odds ratio; other abbreviations see as Table 1 or Supplementary Table 1.

**Supplementary Table 3** Receiver Operating Characteristic Curve Analysis

| Variable | AUC [95%CI] | P-value | Cutoff | Sensitivity (%) | Specificity (%) |
| --- | --- | --- | --- | --- | --- |
| LASr (%) | 0.75[0.62-0.85] | 0.00 | 24.7 | 87.9 | 59.3 |
| LASct (%) | 0.74[0.61-0.84] | 0.00 | -11.6 | 66.7 | 81.5 |
| LASr-4C (%) | 0.70[0.56-0.81] | 0.01 | 23.1 | 93.9 | 55.6 |
| LASct-4C (%) | 0.70[0.56-0.81] | 0.01 | -15.1 | 84.8 | 51.9 |
| E/E′septal | 0.76[0.63-0.86] | 0.00 | 11.1 | 84.9 | 66.7 |
| LASr/E/E′septal (%) | 0.83[0.71-0.92] | 0.00 | 2.1 | 87.9 | 74.1 |
| LASr-4C/E/E′septal (%) | 0.81[0.69-0.90] | 0.00 | 2.0 | 87.9 | 70.4 |

AUC = area under the curve; CI = confidence interval; other abbreviations see as Table 1 or Supplementary Table 1.
