# Supplementary figures and images for: Left atrial reservoir strain combined with E/E' as a better single measure to predict elevated LV filling pressures in patients with coronary artery disease
Source: Cardiovasc Ultrasound. 2020 Apr 25;18:11. doi: 10.1186/s12947-020-00192-4 (PMC7183713; doi:10.1186/s12947-020-00192-4)

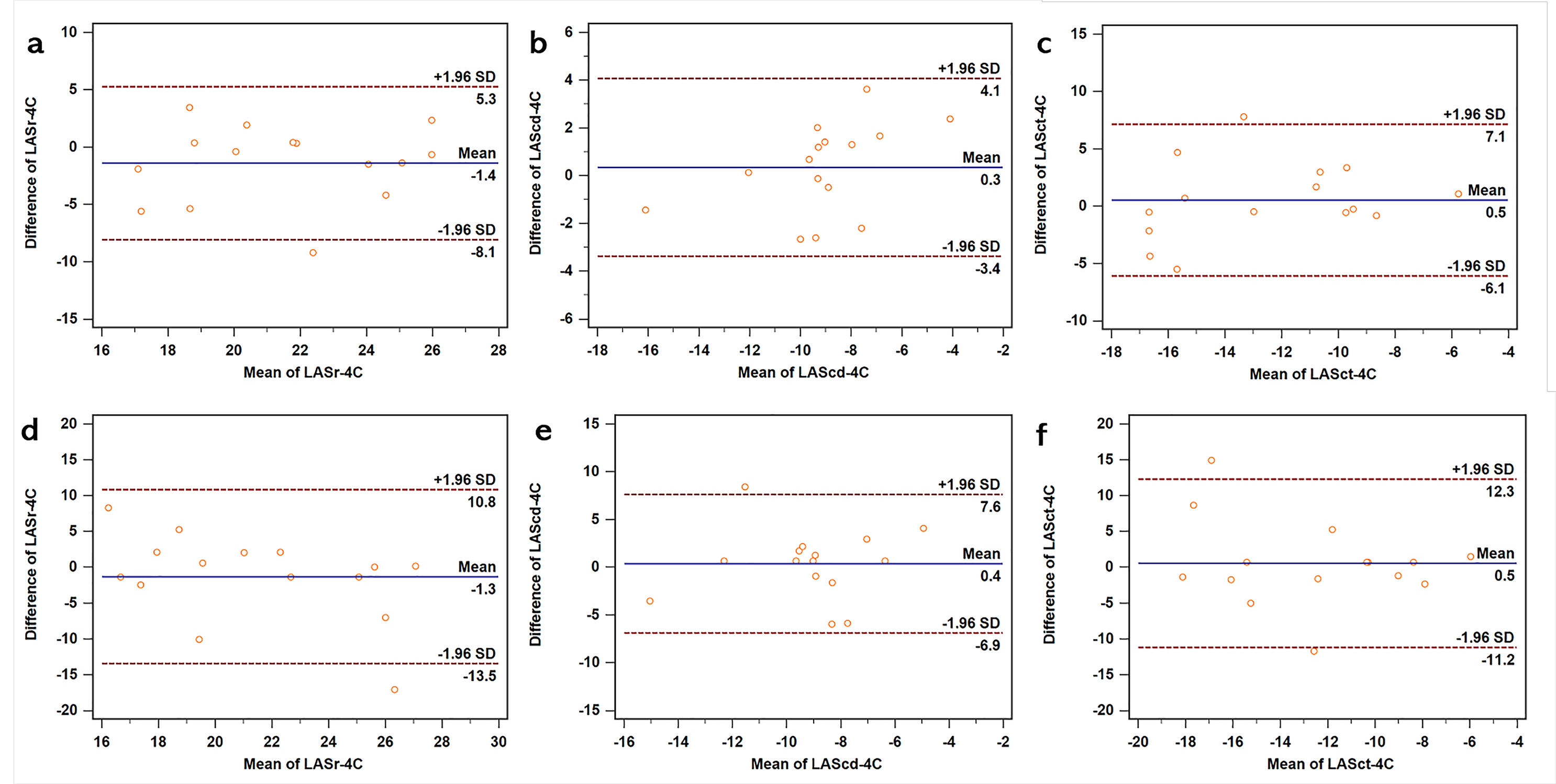

Supplement: Supplementary file 2 — Additional file 2: Figure S1. Bland-Altman analysis for intra-observer and inter-observer variabilities for LA strain measurements of four-chamber view alone. [file 12947_2020_192_MOESM2_ESM.tif]
